# Prevalence of electronic nicotine delivery systems and electronic non-nicotine delivery systems in children and adolescents: a systematic review and meta-analysis

**DOI:** 10.1016/S2468-2667(21)00106-7

**Published:** 2021-07-16

**Authors:** Sze Lin Yoong, Alix Hall, Alecia Leonard, Sam McCrabb, John Wiggers, Edouard Tursan d'Espaignet, Emily Stockings, Hebe Gouda, Ranti Fayokun, Alison Commar, Vinayak M Prasad, Christine Paul, Christopher Oldmeadow, Li Kheng Chai, Bruce Thompson, Luke Wolfenden

**Affiliations:** a School of Health Sciences, Swinburne University of Technology, Hawthorn, VIC, Australia; b School of Medicine and Public Health, University of Newcastle, Callaghan, NSW, Australia; c Priority Research Centre for Health Behaviour, University of Newcastle, Callaghan, NSW, Australia; d Hunter Medical Research Institute, New Lambton Heights, NSW, Australia; e Hunter New England Population Health, Wallsend, NSW, Australia; f School of Rural Medicine, University of New England, Armidale, NSW, Australia; g National Drug and Alcohol Research Centre, University of New South Wales, Randwick, NSW, Australia; h Department of Health Promotion, No Tobacco Unit, World Health Organization, Geneva, Switzerland; i Health and Wellbeing Queensland, Queensland Government, Milton, QLD, Australia; j Centre for Children's Health Research, Institute of Health and Biomedical Innovation Exercise and Nutrition, Queensland University of Technology, South Brisbane, QLD, Australia

## Abstract

**Background:**

There are concerns that the use of electronic nicotine delivery systems (ENDS) and electronic non-nicotine delivery systems (ENNDS) in children and adolescents could potentially be harmful to health. Understanding the extent of use of these devices is crucial to informing public health policy. We aimed to synthesise the prevalence of ENDS or ENNDS use in children and adolescents younger than 20 years.

**Methods:**

In this systematic review and meta-analysis, we undertook an electronic search in five databases (MEDLINE, Web of Science, Cumulative Index to Nursing and Allied Health Literature, Embase, and Wiley Cochrane Library) from Jan 1, 2016, to Aug 31, 2020, and a grey literature search. Included studies reported on the prevalence of ENDS or ENNDS use in nationally representative samples in populations younger than 20 years and collected data between the years 2016 and 2020. Studies were excluded if they were done in those aged 20 years or older, used data from specialist panels that did not apply appropriate weighting, or did not use methods that ensured recruitment of a nationally representative sample. We included the most recent data for each country. We combined multiple national estimates for a country if they were done in the same year. We undertook risk of bias assessment for all surveys included in the review using the Joanna Briggs Institute Critical Appraisal Checklist (by two reviewers in the author list). A random effects meta-analysis was used to pool overall prevalence estimates for ever, current, occasional, and daily use. This study was prospectively registered with PROSPERO, CRD42020199485.

**Findings:**

The most recent prevalence data from 26 national surveys representing 69 countries and territories, with a median sample size of 3925 (IQR 1=2266, IQR 3=10 593) children and adolescents was included. In children and adolescents aged between 8 years and younger than 20 years, the pooled prevalence for ever (defined as any lifetime use) ENDS or ENNDS use was 17·2% (95% CI 15–20, *I*^2^=99·9%), whereas for current use (defined as use in past 30 days) the pooled prevalence estimate was 7·8% (6–9, *I*^2^=99·8%). The pooled estimate for occasional use was 0·8% (0·5–1·2, *I*^2^=99·4%) for daily use and 7·5% (6·1–9·1, *I*^2^=99·4%) for occasional use. Prevalence of ENDS or ENNDS use was highest in high-income geographical regions. In terms of study quality, all surveys scored had a low risk of bias for the sampling frame used, due to the nationally representative nature of the studies. The most poorly conducted methodological feature of the included studies was subjects and setting described in detail. Few surveys reported on the use of flavours or types of ENDS or ENNDS.

**Interpretation:**

There is significant variability in the prevalence of ENDS and ENNDS use in children and adolescents globally by country income status. These findings are possibly due to differences in regulatory context, market availability, and differences in surveillance systems.

**Funding:**

World Health Organization and the Bill & Melinda Gates Foundation.

## Introduction

Electronic nicotine delivery systems (ENDS) and electronic non-nicotine delivery systems (ENNDS) are systems that use devices to heat liquids to create aerosols that are inhaled by the user.^1^ ENDS contain nicotine, whereas ENNDS are labelled as not containing nicotine, although this claim is not always accurate.^1^ There are many forms of ENDS and ENNDS, including e-cigars, e-pipes, and e-hookahs, with e-cigarettes being the most common.^2^ There is mixed evidence describing the health effects of these products in relation to cardiovascular and pulmonary risk.^3^ Reviews based on short-term human, animal, and in-vitro studies suggest that ENDS and ENNDS are associated with increased inflammatory responses and adverse effects on respiratory outcomes.^4^ However, with few long-term safety studies done in humans, the health effects of ENDS and ENNDS remain uncertain.

There have been increases in use of ENDS and ENNDS in children and adolescents (aged <20 years) in some countries.^5^, ^6^ In Canada, for example, national surveys indicate that the prevalence of ENDS and ENNDS use in the past 30 days (from completion of the survey) in high school (aged 12 to 18 years) students increased, from 8% for the years 2013–14 to 26% for 2018–19, with older male (approximately 18 years) students reporting the highest use.^5^ For countries where use of ENDS and ENNDS is increasing, such as New Zealand and the USA,^7^, ^8^ this trend is also accompanied by a decline in cigarette smoking, suggesting that use of ENDS and ENNDS could have contributed to this decline. However, evidence from animal and in-vitro studies show that nicotine, a key component of ENDS, is damaging to the developing brain of children and adolescents.^9^ Further, meta-analyses of prospective cohort studies found that use of e-cigarettes in non-smoking children and adolescents might increase the risk of future cigarette smoking^10^ and use of other addictive substances^3^, ^11^, ^12^; although at present data are sparse and it remains uncertain whether ENDS or ENNDS use is associated with a gateway effect toward use of tobacco and other substances in children and adolescents.

**Research in context**

**Evidence before this study**

We searched MEDLINE and Web of Science using the key words “e-cigarettes”, “electronic nicotine delivery systems (ENDS) and electronic non-nicotine delivery systems (ENNDS)” and “prevalence” from Jan 1, 2016, to Jan 30, 2020, for recent reviews, and PROSPERO for review registrations that sought to describe the global prevalence of ENDS and electronic non-nicotine delivery systems (ENNDS) use in children and adolescents. We identified four reviews that assessed the prevalence of e-cigarette use internationally, with the most recent providing data up to the year 2017. The most comprehensive review included data from only 11 high-income countries. At the time of writing, no reviews were published between 2017 and May, 2021, describing the global prevalence of ENDS and ENNDS use in children and adolescents and none have examined the use of ENNDS specifically.

**Added value of this study**

Nationally representative data from 69 countries and territories are included in this study, which is, to our knowledge, the largest synthesis of data relating to the prevalence of ENDS and ENNDS use in children and adolescents up to Aug 31, 2020. This study identified variability in prevalence of ENDS and ENNDS use in different countries and territories. The use of ENDS and ENNDS was highest in high-income countries and in males for most countries. We found that reported ever or occasional use of ENDS or ENNDS was common in some countries or territories, but the prevalence of daily use was very low across all locations. There were few nationally representative studies assessing the use of non-nicotine delivery systems, flavoured ENDS or ENNDS use, and types of ENDS or ENNDS use, and none of the included studies reported on cannabis use in these devices. This systematic review and meta-analysis provides new insight into the prevalence of ENDS and ENNDS use in children and adolescents in a broader range of countries and territories than previous studies, and highlights gaps where surveillance data are needed to inform policy and practice.

**Implications of all the available evidence**

This systematic review and meta-analysis highlights the importance of reliable and comprehensive data to allow ongoing surveillance on the prevalence of ENDS and ENNDS use in children and adolescents. We provide a synthesis of the patterns of use of ENDS and ENNDS in these age groups to interested parties, including regulators and policy makers to inform public health measures. Specifically, this information will aid the formulation, adoption, implementation, and enforcement of youth-oriented measures and policies to prevent uptake of ENDS and ENNDS. Given the high reported ever or occasional use of ENDS and ENNDS in children and adolescents in some countries and the possibility that usage could grow in other countries in future, public health policies regulating the availability and marketing of such products are needed. Our study also identifies gaps in the assessment of the prevalence of ENDS and ENNDS internationally, particularly in lower-middle-income countries. Internationally, health agencies and governments should seek to improve national and global surveillance systems on the use of ENDS and ENNDS in children and adolescents. Such surveillance mechanisms are crucial to establish the extent, patterns, and trends of ENDS and ENNDS use, and to provide epidemiological data to better support public health policy and practice decisions.

Given these data, there is a need to continue to monitor the prevalence of ENDS and ENNDS to provide insights into the patterns of their use in children and adolescents and to understand the health needs of these populations. The most recent reviews and meta-analyses of ENDS and ENNDS use globally included studies up to the year 2017.^13^, ^14^, ^15^, ^16^ Since then, many countries and territories have included monitoring of ENDS or ENNDS use as part of national surveillance systems.

We aimed to systematically review the literature for the most recent nationally representative estimates of prevalence of ever and current ENDS and ENNDS use between the years 2016 and 2020. In our study, we focussed on children and adolescents and data were reported by sex, when available. Additionally, this study describes the prevalence of ENNDS use, and types and flavours of product used.

## Methods

### Search strategy and selection criteria

This systematic review and meta-analysis was done with reference to guidance provided by the Joanna Briggs Institute^17^, ^18^ on conducting reviews of prevalence and reported consistent with the preferred reporting items for systematic reviews and meta-analyses (PRISMA)^19^ and guidelines for accurate and transparent health estimates reporting (GATHER).^20^

We systematically searched MEDLINE, Web of Science, Cumulative Index to Nursing and Allied Health Literature, Embase, and Wiley Cochrane Library. We developed the search strategy together with an information specialist (DB) using search terms from our previous review^16^ and validated search filters.^21^ Our search terms included “electronic nicotine delivery systems (ENDS), electronic non-nicotine delivery systems (ENNDS)” AND “study design” AND “children OR adolescents” (appendix pp 1–2). Our search was limited to studies published between Jan 1, 2016, and Aug 31, 2020. Reference lists of relevant reviews were also screened.

We also searched relevant funder websites (eg, Centers for Disease Control and Prevention) and liaised with authors (HG and AC) from WHO's No Tobacco Unit to identify relevant data registries. Additionally, we searched for the most recent estimates from national surveys identified within our electronic search. Specifically, the names of relevant surveys were searched in Google Scholar and Google (the first 100 search results screened were sorted by relevance) and the website of the funding body in November, 2020. In instances when there were missing data that were essential for the meta-analysis, we contacted main contacts to obtain additional information.

We included studies if they described the prevalence of ENDS and ENNDS use in children and adolescents (ie, aged <20 years) in the general population. Studies were excluded if they described prevalence in those aged 20 years or older, used data from specialist panels that did not apply appropriate weighting, or did not use methods that ensured recruitment of a nationally representative sample. Cross-sectional, repeat cross-sectional, or longitudinal studies that published data between the years 2016 and 2020 were eligible if they used a probability or census based random sampling method or applied population weights to ensure representativeness to the specific country. For countries where prevalence data from multiple years existed, only the most recent year was retained. When multiple national estimates for a country existed in the same year (eg, two studies done in the USA for the year 2019), all relevant studies were eligible for inclusion in the review. There were no restrictions on peer review status or language.

An information specialist used EndNote version X9.2 software to filter duplicate studies. All screening was done in duplicate with the Covidence software by two reviewers (SLY and AH) and discrepancies resolved via consensus.

### Data analysis

All data were extracted by one reviewer (AH, AL, SM, or SLY) and checked by a second reviewer (AH, AL, or SM) with pre-piloted data extraction forms developed for the study (appendix pp 3–4). These data were extracted: country, sample size, age range, sex, year of data collection, data collection method, sampling procedures, use of sampling weights, measure of ENDS or ENNDS use, type and devices used, flavours used, prevalence and frequency of ENDS or ENNDS use (by ever use [ie, any lifetime use], use in the past 30 days [since survey completion], occasional use [ie, less than daily and more than every 30 days], and daily use [ie, at least once per day]), when reported.

The Joanna Briggs Institute Critical Appraisal Checklist^18^ was used to determine the quality of each survey by one reviewer (AH, AL, SM, or LKC) and checked by a second reviewer (AH, AL, or SM).^22^ The tool consists of nine items examining: (1) sample representativeness, (2) sampling methods, (3) adequacy of sample size, (4) participant and setting descriptions, (5) coverage of sample, (6) objectivity and reliability of measures, (7) appropriateness of statistical analysis, (8) confounding factors identified and accounted for, and (9) objective classification of subpopulations.

Data analyses were done with STATA version 16 with the metaprop and metan packages. Similar to other reviews^23^ to prevent double counting, we included the studies with the most recent or complete data. When multiple national estimates for a country done in the same year existed, these estimates were combined with a fixed effects meta-analysis to provide a single estimate for that country. An overall estimate of prevalence for each outcome across all countries and by income level was generated with the DerSimonian and Laird random effects method of meta-analysis. The Freeman-Tukey double arcsine transformation of prevalence was used to allow for studies with prevalence that were close to zero. Exact 95% CIs were computed. Pooled estimates for weighted prevalence were reported as an absolute percentage, along with 95% CIs. The World Bank Income categories (ie, low, lower-middle, upper-middle, and high-income)^24^ were used to categorise country income level. Study findings were described narratively, by frequency of use, sex, flavour type, and type of ENDS or ENNDS used. This study was prospectively registered (PROSPERO CRD42020199485).

### Role of the funding source

WHO funded the review and was involved in the development of the study's aims. Members of the WHO team are also authors on this manuscript (HG, RF, AC, and VMP) and provided input into the manuscript and final approval consistent with authorship guidelines. The funding source was not involved in data collection and analysis.

## Results

**Figure 1:**
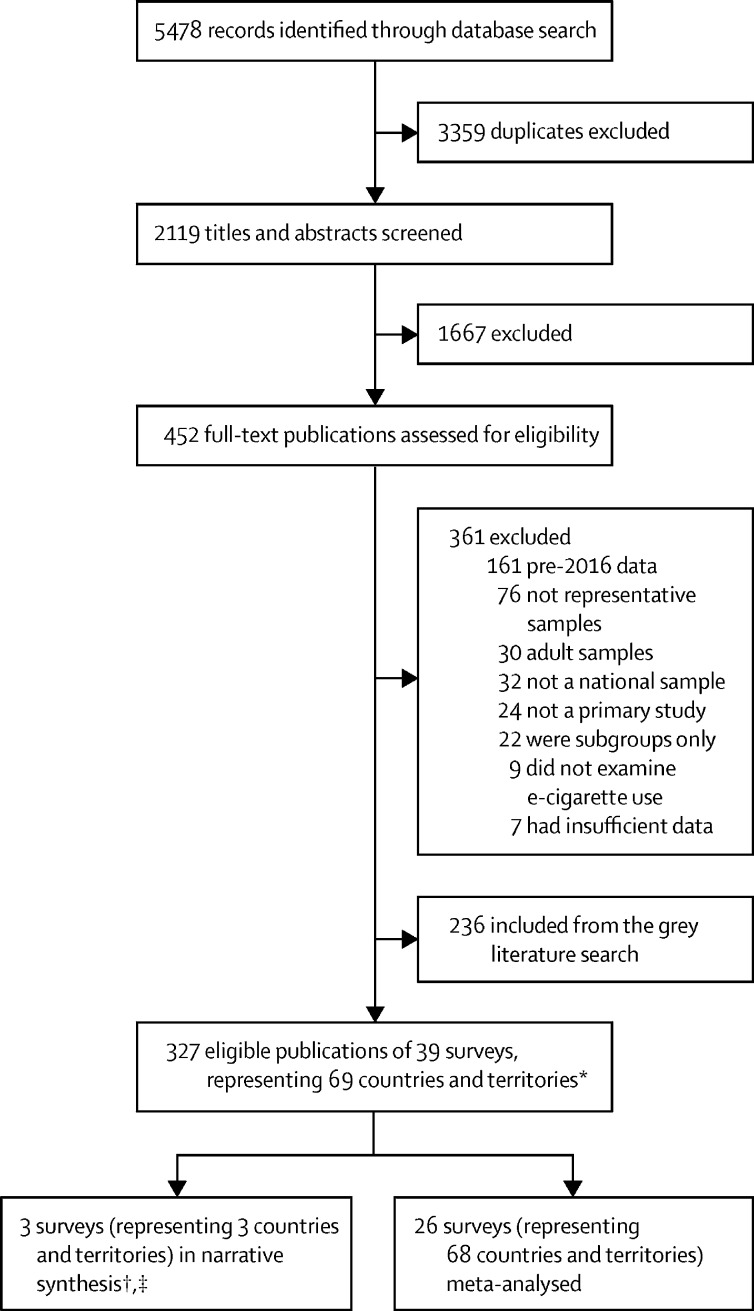
Study selection *13 of the 39 eligible surveys were not included in the meta-analyses as they did not represent the most recent data for a country and were thus superseded by another eligible survey. †One survey done in Kazakhstan was not included in the meta-analyses due to insufficient data. ‡Two surveys were included in the narrative synthesis only as they described use by flavours (the Youth Insight Survey done in New Zealand) and types and devices (the Tobacco & E-Cigarette Survey among Malaysian Adolescents [TECMA] done in Malaysia). They were not included in the meta-analysis as they did not provide the most recent estimates of electronic nicotine delivery systems and electronic non-nicotine delivery systems use and were superseded by a more recent survey.

Of the 5478 published articles identified, 91 were included in our systematic review (figure 1). An additional 236 information sources were identified from the grey literature describing relevant national surveys including ENDS or ENNDS use by children and adolescents. A total of 327 documents reported on 39 national surveys (representing 69 countries and territories) were included, reporting Hong Kong separately (as a special administrative region of China) and Guam (as a territory of the USA). Surveys reporting on the UK were included, as were those reporting separately on Scotland, England, and Wales (appendix p 5). 26 surveys from both published and grey literature sources provided the most recent estimates on ENDS or ENNDS use from 68 countries and territories and were included in the meta-analysis. The median sample size was 3925 participants (interquartile 1=2266 participants and interquartile 3=10 593 participants). One country (Kazakhstan) did not have sufficient data to be included in the meta-analysis, so findings were narratively described. Four published surveys (from three countries) reported on flavoured ENDS or ENNDS use (one of these was not included in the main meta-analysis).^25^, ^26^, ^27^, ^28^ Three published surveys (from three countries and territories) reported on types and devices of ENDS or ENNDS use, including one not included in the meta-analysis.^26^, ^29^, ^30^

50 studies, primarily from the grey literature, included data from global surveillance systems (42 studies from the Global Youth Tobacco Survey and eight studies from the Health Behaviour in School-Aged Children survey; appendix pp 7–10). The age of participants ranged from 8 years to 15 years (in the Health Survey for England survey) and 15 to 20 years (in the Truth Longitudinal Cohort survey). None of the 39 studies included participants younger than 8 years and six studies included participants older than 18 years. All studies used self-reported surveys to assess ENDS or ENNDS use, and all except one^31^ referred to e-cigarettes (sometimes together with e-shisha, e-hookah, and e-pipes).

**Figure 2:**
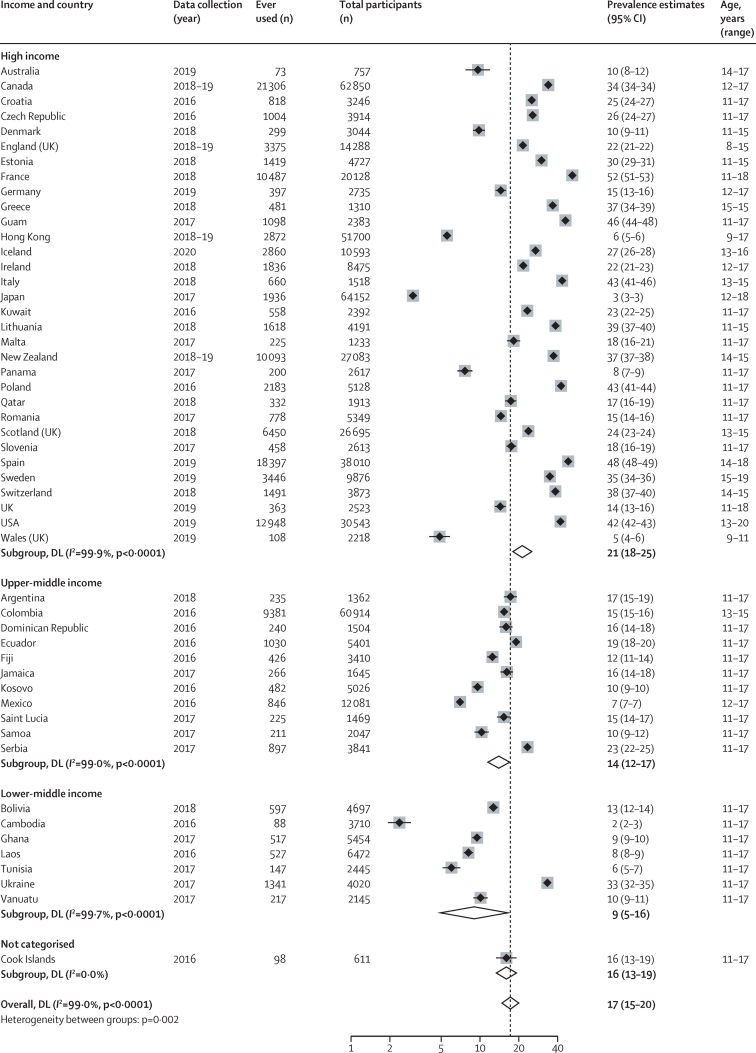
Prevalence of ever use of electronic nicotine delivery systems and electronic non-nicotine delivery systems Locations presented by World Bank income classification. Weights and between subgroup heterogeneity tests are from random effects models. DL=DerSimonian‐Laird method of assessing heterogeneity.

51 countries and territories from high-income (n=32), upper-middle-income (n=11), lower-middle-income (n=7), and uncategorised (n=1 [Cook Islands]) countries reported on ever use of ENDS or ENNDS in children and adolescents younger than 20 years. Prevalence estimates for ever use ranged from 2% (95% CI 2–3) in Cambodia to 52% (51–53) in France (figure 2). Pooled estimates across all available countries were 17% (15–20). In all 11 countries that reported prevalence by sex, except for Iceland, prevalence in males was higher than in females for the particular country (table).

**Table:** Prevalence estimates for use of electronic nicotine delivery systems and electronic non-nicotine delivery systems

	**n**	**Prevalence, % (95% CI)**	**Female, n**	**Male, n**	**Female prevalence, % (95% CI)**	**Male prevalence, % (95% CI)**
**Ever use**						
England (UK)	14 288	21·5 (20·9–22·2)	7236	6842	23·0 (22·0–24·0)	27·0 (25·9–28·1)
Estonia	4727	30·0 (28·7–31·3)	2357	2370	24·0 (22·3–25·8)	36·0 (34·1–37·9)
Germany	2735	14·5 (13·2–15·9)	1321	1413	10·3 (8·8–12·1)	18·5 (16·5–20·6)
Iceland	10 593	27·0 (26·2–27·9)	5200	5222	26·0 (24·8–27·2)	26·0 (24·8–27·2)
Ireland	8475	21·7 (20·8–22·6)	4594	3881	18·0 (16·9–19·1)	26·0 (24·6–27·4)
Japan	64 152	3·0 (2·9–3·2)	29 570	34 582	2·0 (1·8–2·1)	4·1 (3·9–4·3)
Lithuania	4191	38·6 (37·1–40·1)	2058	2133	33·0 (31·0–35·1)	44·3 (41·9–46·1)
Mexico	12 081	7·0 (6·6–7·5)	5920	6161	5·0 (4·5–5·6)	8·0 (7·4–8·7)
New Zealand	27 083	37·3 (36·7–37·8)	13 635	13 002	33·4 (32·7–34·2)	40·6 (39·7–41·4)
Scotland (UK)	26 695	23·8 (23·3–24·3)	11 449	11 449	22·0 (21·3–22·8)	29·0 (28·2–29·8)
Switzerland	3873	38·5 (37·0–40·0)	1991	1882	30·9 (28·9–33·0)	46·5 (44·3–48·8)
**Current use**						
England (UK)	13 191	6·0 (5·6–6·4)	6704	6277	5·0 (4·5–5·6)	7·0 (6·4–7·7)
Germany	2735	4·1 (3·4–4·9)	1321	1413	2·7 (2·0–3·8)	5·0 (4·3–6·6)
Ireland	8475	8·4 (7·8–9·0)	4594	3881	7·0 (6·3–7·8)	10·0 (9·1–11·0)
Japan	64 152	0·9 (0·8–1·0)	29 570	34 582	0·5 (0·4–0·6)	1·0 (1·2–1·4)
Lithuania	4191	18·1 (16·9–19·3)	2058	2133	15·0 (13·5–16·6)	21·0 (19·3–22·8)
Malaysia	27 497	9·3 (8·9–9·6)	14 362	13 135	2·5 (2·3–2·8)	17·0 (16·0–17·3)
Mexico	12 068	1·1 (0·9–1·3)	5913	6155	0·6 (0·4–0·8)	2·0 (1·7–2·4)
South Korea	60 040	2·4 (2·3–2·6)	29 577	30 463	1·1 (1·0–1·2)	4·0 (3·5–4·0)
Switzerland	3873	16·0 (14·9–17·2)	1991	1882	12·7 (11·3–14·2)	20·0 (17·8–21·4)
Taiwan	44 289	2·7 (2·6–2·9)	21 338	22 951	1·5 (1·3–1·6)	4·0 (3·7–4·2)
USA	49 039	22·8 (22·4–23·2)	6464	6183	33·5 (32·4–34·7)	32·0 (30·9–33·2)
**Daily use**						
Finland	153 142	2·1 (2·1–2·2)	78 963	73 922	0·9 (0·9–1·0)	3·4 (3·2–3·5)
Japan	64 152	0·1 (0·1–0·1)	29 570	34 582	0·1 (0·1–0·2)	0·1 (0·1–0·1)
New Zealand	26 532	3·1 (2·9–3·4)	13 429	12 668	2·4 (2·1–2·7)	3·7 (3·4–4·0)
USA	31 701	4·3 (4·1–4·6)	6464	6183	6·4 (5·8–7·0)	7·9 (7·3–8·6)
**Occasional use**						
England (UK)	13 191	4·0 (3·7–4·4)	6704	6277	4·0 (3·6–4·5)	4·0 (3·5–4·5)
New Zealand	27 354	12·0 (11·6–12·4)	14 987	13 446	10·8 (10·3–11·3)	12·8 (12·2–13·4)

Data given for studies that disaggregated results by sex. Ever use is defined as any lifetime use. Current use is defined as use in past 30 days. Occasional use is defined as less than daily and more than every 30 days

**Figure 3:**
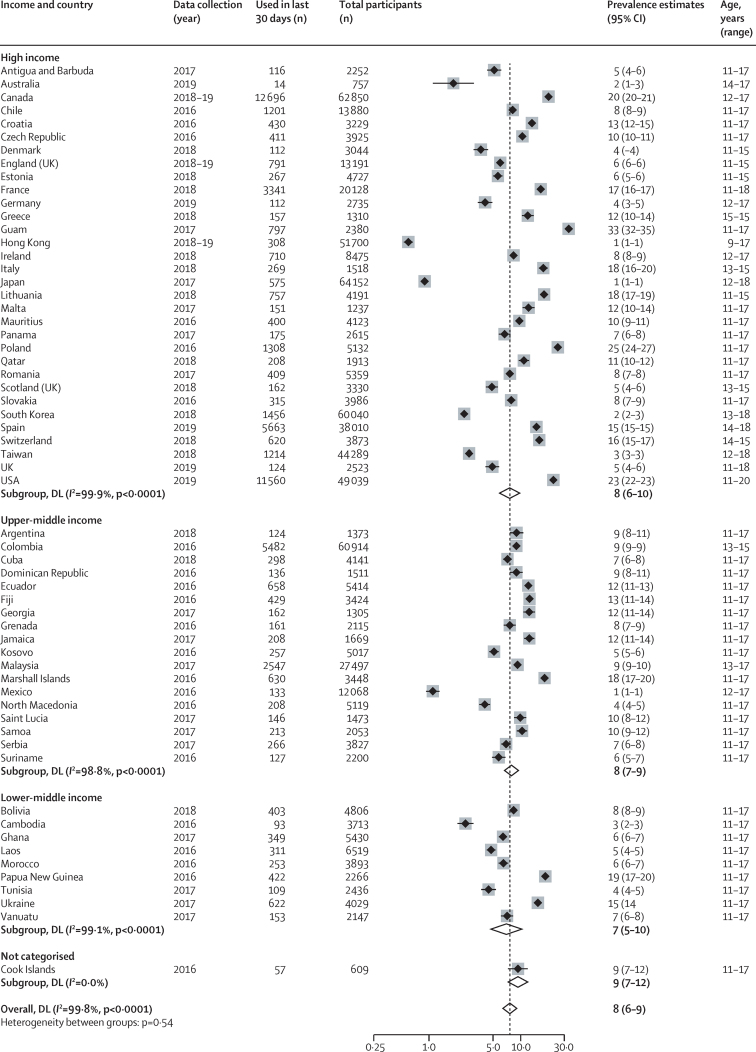
Prevalence of current use of electronic nicotine delivery systems and electronic non-nicotine delivery systems by World Bank income classification Locations presented by World Bank income classification. Weights and between subgroup heterogeneity tests are from random effects models. DL=DerSimonian‐Laird method of assessing heterogeneity.

60 countries and territories from high-income (n=32), upper-middle-income (n=18), lower-middle-income (n=9), and uncategorised (n=1) countries reported on current use (most frequently defined as use in the past 30 days since survey completion [figure 3]). Prevalence estimates ranged from 1% (95% CI 1–1) for Hong Kong, Japan, and Mexico, to 33% (32–35) for Guam. Pooled estimates across all available countries and territories were 8% (6–9). 11 countries reported on prevalence by sex, with prevalence in males higher than in females across all included study sites, except for the USA (table).

**Figure 4:**
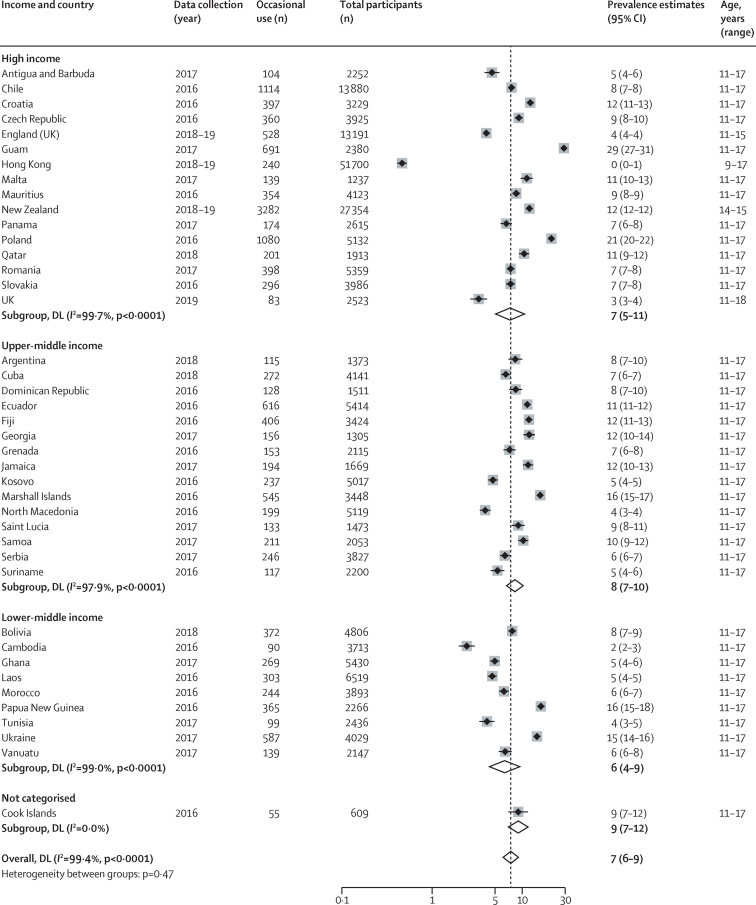
Prevalence of occasional use of electronic nicotine delivery systems and electronic non-nicotine delivery systems Locations presented by World Bank income classification. Weights and between subgroup heterogeneity tests are from random effects models. DL=DerSimonian‐Laird method of assessing heterogeneity.

41 countries and territories from high-income (n=16), upper-middle income (n=15), lower-middle income (n=9), and uncategorised (n=1) countries reported on occasional use of ENDS or ENNDS (figure 4). Prevalence estimates ranged from 0% (95% CI 0–1) in Hong Kong to 29% (27–31) in Guam. The pooled estimate across all study sites was 7% (6–9). Only England and New Zealand reported occasional ENDS or ENNDS use by sex (table).

**Figure 5:**
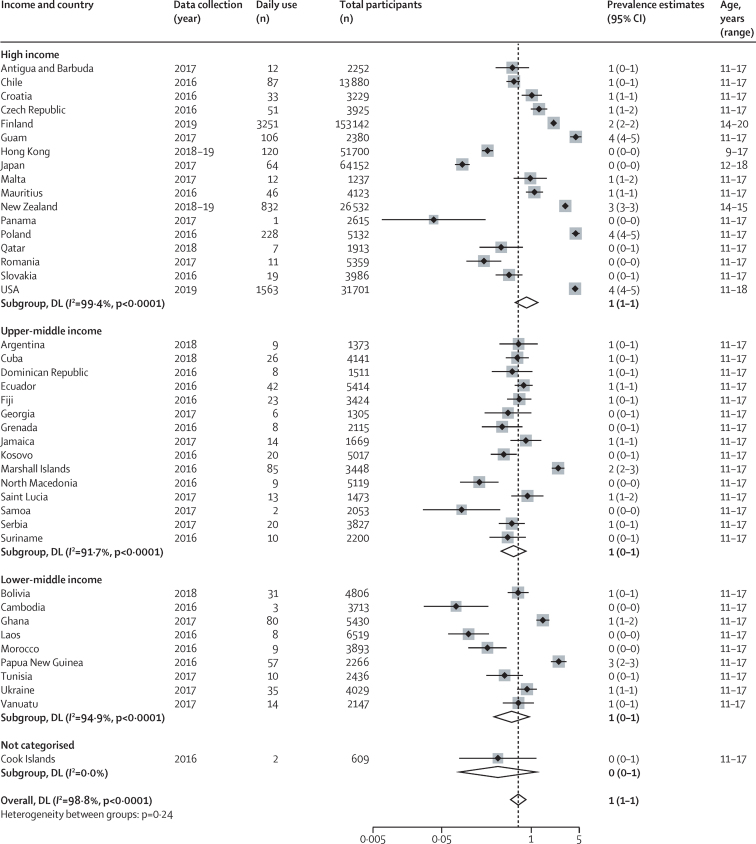
Prevalence of daily use of electronic nicotine delivery systems and electronic non-nicotine delivery systems Locations presented by World Bank income classification. Weights and between subgroup heterogeneity tests are from random effects models. DL=DerSimonian‐Laird method of assessing heterogeneity.

42 high-income countries and territories (n=17), upper-middle-income (n=15), lower-middle-income (n=9), and uncategorised (n=1) countries reported on daily use (figure 5). Prevalence estimates ranged from 0% (95% CI 0–0) for Panama, Cambodia, Hong Kong, Japan, Laos, Morocco, Romania, and Samoa, to 4% (4–5) for the USA, Guam, and Poland. The pooled estimate across all available study sites was 1% (1–1). Four countries reported prevalence by sex, with prevalence in males higher than in females in Finland, New Zealand, and the USA, but not in Japan where prevalence was very low (table).

Five countries reported on ENNDS use (appendix p 11). Three of these countries reported on current ENNDS use, with prevalence estimates ranging from 0·9% (95% CI 0·6–1·4) in the UK (including England, Scotland, and Wales) to 11% (11·1–11·5) in Canada. Two countries reported data for daily ENNDS use, with estimates similar between Canada (2%; 1·9–2·1) and the USA (2%; 1·8–2·2). Only Canada reported prevalence of occasional ENNDS use (as 9% [9·1–9·6]). Estimates for ENDS use (in which only nicotine e-cigarettes were used) were available from six countries (Austria, Canada, Italy, Spain, the UK, and the USA) and are presented in the appendix (p 12).

Three countries (New Zealand, the UK, and the USA) reported on whether participants used flavoured products. In New Zealand, 68% of ever ENDS or ENNDS users reported using a sweet flavour in their last use in the 2018 Youth Insights Survey.^27^ Similarly, 63% of current users reported using sweet flavours. In the UK, in ENDS or ENNDS users aged between 11 and 18 years, 45% reported using fruit flavours.^26^

The US 2018 National Youth Tobacco Survey (which provides the most recent estimate for flavoured products) found that an estimated 72·2% (95% CI 69·1–75·1) of high school students (aged 14 to 18 years) who were current exclusive users of ENDS or ENNDS used flavoured products.^28^ In middle school (aged 11 to 14 years) students in the same survey, 59% (54·8–63·4) reported use of flavoured ENDS or ENNDS. The most frequently reported flavour categories were fruit (high school: 66% [62·4–69·5]; middle school: 68% [62·6–72·5]), and menthol or mint flavour (high school: 57% [53·3–61·3]; middle school: 31% [25·6–37·2]).

The US monitoring the future study found that 19% of eighth, tenth, and 12th graders (approximately between 13 years and 18 years) reported using JUUL (Juul Labs, San Francisco, CA, USA), a brand of ENDS and ENNDS, in the past 30 days (since completing the survey).^25^ In current users, the flavours used by eighth grade students were mango (33·5%; 95% CI 28·7–38·7), mint (29·2%; 22·7–36·8), and other fruit (16·0%; 12·1–20·9). In tenth graders who are current users, mint (43·5%; 37·1–50·1), mango (27·3%; 23·1–31·9), and other fruit (10·8%; 8·1–14·1) were most popular.^25^ In 12th graders who are current users, mint (47·1%; 41·5–52·8), mango (23·8%; 18·8–29·7), and other fruit (8·6%; 6·0–12·0) were also the most popular.

In our review, only one country reported on types of ENDS or ENNDS used. In Germany, 9·2% (95% CI 8·9–9·4) of 12–17 year olds were classified as ever users of e-shisha and 1·8% (0·8–2·1) were current users of e-shisha.^30^

Two countries reported on devices used. A 2016 survey in Malaysia^29^ found that 33·7% (95% CI 29·9–37·8) of adolescents who ever used ENDS or ENNDS used the modular system or Vape-mods and 13·4% (11·2–16·0) used disposable ENDS or ENNDS. In male adolescents, 34·9% (30·8–39·3) used modular systems and 13·1% (10·8–15·8) used disposable ENDS or ENNDS. However, in female adolescents, a rechargeable ENDS or ENNDS kit containing a refillable liquid was most frequently used (29·1% [19·4–41·2]). Such findings were similar for current users for rechargeable e-cigarette kits.

The Action on Smoking and Health Smokefree Great Britain Youth 2019 survey^26^ reported that in current ENDS or ENNDS users, 5% used a disposable, non-rechargeable e-cigarette; 18% used an ENDS or ENNDS kit that is rechargeable with replaceable pre-filled cartridges; and 62% used an e-cigarette that has a tank or reservoir filled with liquids.

Risk of bias was assessed for 27 included surveys (appendix pp 13–14). Risk of bias was highest for the Alcohol & Drug use Among Students in the Skolelevers drogvanor (CAN) school survey, which had only three of the nine items classified as a low risk of bias. All studies had a low risk of bias for sampling frame because of the nationally representative nature of the studies. The subjects and setting described in detail was most poorly adhered to, with six of the 27 surveys reporting a high risk of bias for this item.

## Discussion

This systematic review and meta-analysis highlights that nationally representative prevalence data for ENDS or ENNDS use in children and adolescents is scarce globally. Our systematic review identified differences in prevalence of use by country or territory, and income.^24^ The prevalence of ever use of ENDS or ENNDS in children and adolescents was more than 40% in several high-income countries, including France, Spain, Guam, Italy, Poland, and the USA. Over 20% of children and adolescents in Guam, Poland, and the USA reported being current users of ENDS or ENNDS. Daily use was less than 1% for most countries and territories analysed, with Guam, Poland, and the USA reporting the highest prevalence.

Our study has several strengths. We did a comprehensive systematic review and searched extensively across electronic databases and grey literature with no language restrictions. We also consulted with authors from WHO to identify literature that was not readily located. We contacted authors of original publications for additional data to enable the inclusion of these studies in the meta-analysis. Additionally, we checked all relevant websites for data release and sourced all related data linked to a publication to support extraction. Although there was high heterogeneity of pooled estimates, many of the included surveys used standardised data collection methods and measures.

Limitations of our study need to be acknowledged. First, current use of ENDS or ENNDS was frequently defined as use in the past 30 days (since completing the survey). Although use in the past 30 days is considered a reasonable proxy of regular use,^32^, ^33^ this measure includes children and adolescents who might not have progressed to regular use. This limitation also applies to the definition of ever users, who are likely to include only single-time users. Additionally, all studies used self-reported measures to assess ENDS or ENNDS use. As such, our findings might have been affected by potential misreporting and are likely to represent an underreporting of actual use. Second, because of the small number of countries and territories included, the pooled analysis might not be representative of global prevalence. As such, our findings are unlikely to be representative of low-income countries. However, our study provides a reasonable estimate for countries included in this review, in particular high-income countries. Third, given this is a rapidly emerging area of research, it is possible that we could have missed updated estimates published since our search was done. For example, the 2020 US National Youth Tobacco Survey^34^ was published outside of our search period (December, 2020). However, this study did not include denominators and 95% CIs, which precluded inclusion in our meta-analysis and review. The survey reports that 20% of high school students and 5% of middle school students were current ENDS or ENNDS users.^34^ Such findings, in contrast to previous research, show a decline in use of ENDS or ENNDS in US children and adolescents, suggesting that there is a need to continue to monitor prevalence to provides data for trends of use in these age groups. Fourth, over a fifth of surveys (22%) did not provide adequate detail of sample characteristics. Consequently, understanding the generalisability of study results is challenging. Fifth, despite the probable differences by age,^28^ we were unable to report pooled prevalence by age groups because only a few surveys reported on the prevalence of ENDS or ENNDS use by age, with inconsistent cutoff points. Lastly, our review did not describe the use of substance included within ENDS and ENNDS (ie, cannabis) as it was beyond the scope of the review.

There have been few global reviews of the prevalence of ENDS or ENNDS use. A previous review^16^ summarised regional and national prevalence of ENDS or ENNDS use in children and adolescents in 2013–15. This review included estimates of ever and current use of ENDS or ENNDS from 11 high-income countries. Our review included updated estimates for ten of the 11 countries (except for Hungary). For most countries (except for ever users in Ireland and current users in the USA), increases in use of ENDS or ENNDS were observed.^35^, ^36^

The highest pooled prevalence of ENDS or ENNDS use was observed in high-income countries and territories, with the lowest pooled prevalence observed in lower-middle-income countries and territories, consistent with contemporary studies in adults.^37^, ^38^ Children and adolescents in high-income countries are likely to have higher disposable income than those in other countries. As such, they are targeted by and exposed to aggressive marketing of these products, which could explain the higher experimentation and use of these products than for children and adolescents in other countries.^39^ There is substantial variability in the regulations, marketing, and availability of ENDS or ENNDS internationally, which could also account for the between country variation observed.^40^ Many countries have introduced minimum age policies for purchasing of ENDS or ENNDS to restrict their use in children and adolescents.^41^ However, these policies are inconsistent (ie, some do not restrict purchasing of non-nicotine or flavoured products) and are challenging to enforce given the wide availability of such products online. Further, the increase in advertising and promotion of these products, including use of online influencers, tobacco and related sponsorships, and use of technology and sleek designs, has also been suggested as other reasons for the potentially increasing popularity in children and adolescents in high-income countries.^42^, ^43^, ^44^

This systematic review and meta-analysis found that daily use of ENDS or ENNDS in children and adolescent occurs in fewer than 1% of national samples of children and adolescents in most countries. As there is little data to inform on the different harms associated with different frequencies of ENDS or ENNDS use, the high prevalence of ever, occasional, and current use continues to be a cause of concern, particularly for non-smokers.^10^, ^45^, ^46^, ^47^ When reported, there was a higher prevalence of use in males than females for most outcomes. These findings are similar to those reported for tobacco use generally,^48^ and suggest that there might be a need to consider tailoring of public health strategies to address this disparity.

Only a few countries assessed the prevalence of ENNDS use (ie, Canada, Italy, Spain, the UK, and the USA) and flavours of ENDS and ENNDS (ie, New Zealand, the UK, and the USA). Some studies suggest that children and adolescents might perceive ENNDS to be less harmful than nicotine containing devices and unflavoured devices.^33^, ^49^ However, apart from nicotine, there are other substances contained in the aerosol of ENDS and ENNDS that could be harmful to health.^50^

The data reported here need to be considered in the context of wider tobacco control and the regulatory frameworks of different countries and existing tobacco use. There is some research suggesting the efficacy of such products for cessation in adult smokers^51^ and epidemiological data indicating a concurrent decline in cigarette smoking in children and adolescents in countries where ENDS or ENNDS use is increasing,^7^, ^8^ with high rates of dual use (ie, concurrent use of tobacco and ENDS or ENNDS) in adolescents.^52^ Strategies to prevent the use of ENDS or ENNDS by non-smoking children and adolescents are warranted given that use of ENDS or ENNDS affords no health benefits in this group, might cause harm, and might increase the risk of future tobacco use.^10^ The potential of ENDS or ENNDS to help dual users to transition from the use of tobacco or as cessation aids in adolescents already using tobacco is unclear. Although ENDS and ENNDS might potentially support adults to quit smoking in some contexts,^51^ unlike adults, most adolescents do not use ENDS or ENNDS as an alternative to cigarettes.^53^ Modelling of the population effects of changes in ENDS or ENNDS use in the UK and the USA suggests that there could be either net benefits or harms depending on the extent of ENDS or ENNDS uptake and the regulatory environment.^54^, ^55^

Although monitoring data for the prevalence of ENDS or ENNDS use in children and adolescents has increased globally, there are still considerable data gaps. Further, there is an absence of nationally representative information on use of ENNDS, flavours, and types of ENDS or ENNDS use. There is a need for more routine inclusion of standardised items assessing ENDS or ENNDS in surveillance systems (eg, the Global Youth Tobacco Survey), or other national tobacco, drug and alcohol, or health behaviour surveys, particularly for low-income countries. This inclusion is recommended to enable greater international coverage and monitoring of use over time to provide important data to support public health policy and practice decisions. Countries should consider adopting policies and other measures that restrict access to ENDS or ENNDS, particularly for children and adolescents. As the evidence develops, countries must continuously monitor, update, and enforce regulations as appropriate to limit the potential harms of ENDS and ENNDS in children and adolescents.

For more on the **Covidence software** see https://www.covidence.org/

## Data sharing

The data analysed during the current study are available from the corresponding author on reasonable request.

## Declaration of interests

We declare no competing interests.
