# Supplementary material for: Prevalence of electronic nicotine delivery systems and electronic non-nicotine delivery systems in children and adolescents: a systematic review and meta-analysis
Source: Lancet Public Health. 2021 Jul 16;6(9):e661–73. doi: 10.1016/S2468-2667(21)00106-7 (PMC8390387; doi:10.1016/S2468-2667(21)00106-7)
Supplement: Supplementary appendix [file mmc1.pdf]

THE LANCET
Public Health

Supplementary appendix

This appendix formed part of the original submission and has been peer reviewed. We post it as supplied by the authors.

Supplement to: Yoong SL, Hall A, Leonard A, et al. Prevalence of electronic nicotine delivery systems and electronic non-nicotine delivery systems in children and adolescents: a systematic review and meta-analysis. *Lancet Public Health* 2021; published online July 15. http://dx.doi.org/10.1016/S2468-2667(21)00106-7.

Supplementary Material

Appendix A: Search strategy for the review in MEDLINE

Database(s): **Ovid MEDLINE(R) and Epub Ahead of Print, In-Process & Other Non-Indexed Citations and Daily** 1946 to August 31st 2020

#	Searches
1	Electronic Nicotine Delivery Systems/
2	e-cig*.mp.
3	(electr* adj2 cig*).mp.
4	(electr* adj2 nicotine).mp.
5	(nicotine adj2 delivery).mp.
6	((ENND* or END*) adj3 nicotine).mp.
7	(vape or vaping).mp.
8	e-liquid.mp.
9	e-nicotine.mp.
10	electronic hookah*.mp.
11	e-hookah*.mp.
12	e-shisha*.mp.
13	e-waterpipe*.mp.
14	1 or 2 or 3 or 4 or 5 or 6 or 7 or 8 or 9 or 10 or 11 or 12 or 13
15	Cross-Sectional Studies/
16	Prevalence/
17	(cross-sectional or prevalence or transversal).tw,kw.
18	Cohort Studies/
19	Longitudinal Studies/
20	Follow-Up Studies/
21	Prospective Studies/
22	Retrospective Studies/
23	cohort.tw,kw.
24	longitudinal.tw,kw.
25	prospective.tw,kw.
26	retrospective.tw,kw.
27	Epidemiologic Studies/

28	Case-Control Studies/
29	Control Groups/
30	Matched-Pair Analysis/
31	((case* adj5 control*) or (case adj3 comparison*) or control group*).tw,kw.
32	evaluation study/
33	Evaluation Studies as Topic/
34	Program Evaluation/
35	Validation Studies as Topic/
36	((pre- adj5 post) or (pretest adj5 posttest) or (program* adj6 evaluat*)).tw,kw.
37	(effectiveness or intervention*).tw,kw.
38	tobacco survey*.mp.
39	15 or 16 or 17 or 18 or 19 or 20 or 21 or 22 or 23 or 24 or 25 or 26 or 27 or 28 or 29 or 30 or 31 or 32 or 33 or 34 or 35 or 36 or 37 or 38
40	adolescent/ or child/
41	Young Adult/
42	(child* or teen* or youth* or juvenile* or "pre adult*").mp.
43	(young adj2 (adult* or person* or individual* or people* or population* or man or men or wom?n)).mp.
44	(emerging adj2 (adult* or person*)).mp.
45	student*.mp.
46	40 or 41 or 42 or 43 or 44 or 45
47	14 and 39 and 46
48	animals/ not humans/
49	47 not 48
**50**	**limit 49 to yr="2016 -Current"**

Appendix B: Variable extracted in data extraction form and description

Variable name	Description
Citation	Inlude first author name, publication year and full title
Survey	The name of the specific survey that this study was a part of
Sample weights	Whether the sample was weighted to ensure representative of national sample.
Date range of data collection	Specific date range when data was collected.
Funding body	Name of the funding body.
Sponsor organisation	Name of sponsor organisation.
Country	Country (specific state, city of count if relevant)
Mode data collect	Mode of data collection by online, paper and pencil or face to face
Sample procedure	Enter the sampling procedures used.
Other sampling information	Enter any details about additional restrictions that were placed on the sample, such as only one third of the total sample received the e-cig questions, or restricted to just current users.
Survey item e-cig use	The wording of the survey item used to assess e-cig use.
Response options e-cig use	The response options that correspond to the item assessing e-cig use.
How current defined	Detail how current e-cig user was defined.
How occassional defined	Detail how ENDS occasional e-cig user was defined.
How use 30 days defined	Detail how ENDS use in last 30 days was defined.
Survey item ENNDS use	The wording of the survey items used to assess ENNDS use.
Response options ENNDS use	The response options that correspond to the item assessing ENNDS.
Flavour item	The wording of the survey items used to assess flavours.
Weights	Whether the sample was weighted to ensure representative.
Weight description	List the characteristics that the sample was weighted by. Make sure they are listed in order of the weights listed below.

Type of ENDS	The type of ENDS assessed.
Year of Data Collection	
Analysed	Total number of participants analysed (i.e. denominator)
Sample	The specific sub-sample that the outcome was assessed in. Please use the exact categories listed. There should be one row per outcome, sub-sample and flavour.
Flavour_content	The specific flavour that the outcome was assessed in.
Male (%)	Percentage of males in sample.
Female (%)	Percentage of females in sample.
M (n)	Number of males assessed.
F (n)	Number of females assessed.
Age range	Age range.
total_n	Number of e-cig users.
Total_Prevalence	Percentage of all e-cig users.
total_LCL	Lower confidence intervals
total_UCL	Upper confidence intervals
male_n	Number of male e-cig users.
male_Prevalence	Percentage of male e-cig users.
male_LCL	Lower confidence intervals for male users.
male_UCL	Upper confidence intervals for male users.
female_n	Number of female e-cig users.
female_Prevalence	Percentage of female e-cig users.
female_LCL	Lower confidence intervals for female users.
female_UCL	Upper confidence intervals for female users.

Supplementary Figures

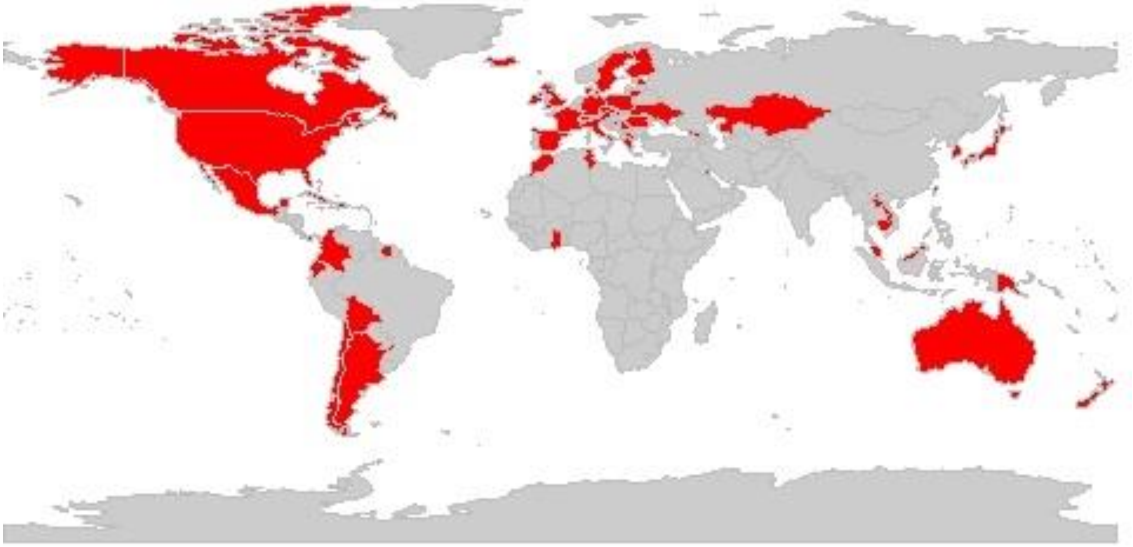

Figure S1. Countries and territories with national prevalence estimates of electronic nicotine delivery system and electronic non-nicotine delivery system use from 2016-2020

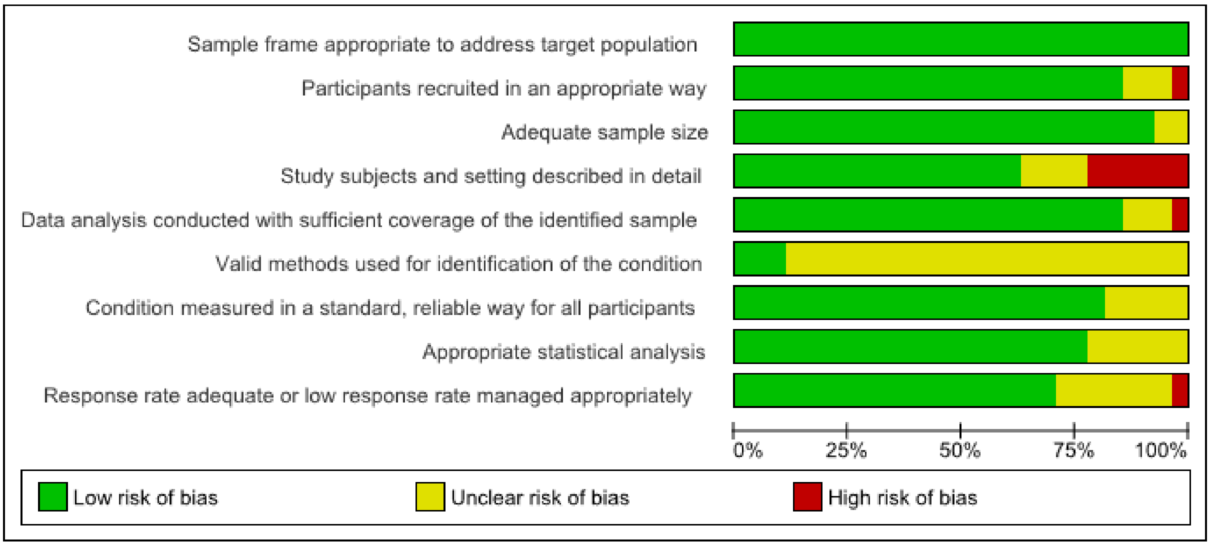

Figure S2. Summary of risk of bias for surveys included in the synthesis

Supplementary Tables

Table S1. Study characteristics of eligible surveys by country

Survey	Country	Year of data collection	N (analysed)	Sampling procedure	Sex	Data collection modality	ENDS/ENNDS use reported	Age range	Included in meta-analysis
Australian Secondary School Students Alcohol and other Drugs Survey (ASSAD)[1]	Australia	2017	19115	Cluster-based sampling.	M: 47%<br>F: 54%	Pen-and-paper	Ever use<br>Current	12-17 years	N, superseded by later survey
National Drug Strategy Household Survey[2]	Australia	2019	757	Probability sampling.	M: 53%<br>F: 47%	Mixed	Ever use<br>Current	14-17 years	Y
European School Survey Project on Alcohol and Other Drugs (ESPAD)[3]	Austria	2019	10279	Random sampling.	M: 52%<br>F: 38%	Online	Ever use ENDS only<br>Current ENDS only	14-17 years	Y
Canadian Student Tobacco, Alcohol and Drugs Survey (CSTADS)[4]	Canada	2018-19	62850	Stratified random sampling covering 10 provinces.	NR	Telephone	Ever use<br>Current<br>Daily – ENDS only and ENNDS only<br>Occasional – ENDS only and ENNDS only<br>Ever – ENDS and ENNDS only<br>Current – ENDS and ENNDS only	12-17 years	Y
Health Survey for England[5]	England (United Kingdom)	2018	1097	Probability sampling.	NR	Pen-and-paper	Ever use	8-15 years	Y
Smoking, Drinking and Drug Use among Young People in England[6]	England (United Kingdom)	2018-2019	13191	Probability sampling.	NR	Pen-and-paper	Ever use<br>Current<br>Occasional<br>Regular	11-15 years	Y
National School Health Promotion Survey[7]	Finland	2019	153142	Population-based sampling.	NR	Online	Daily	14-20 years	Y
EnCLASS (HBSC and ESPAD)[8]	France	2018	20128	Random sampling.	NR	Online	Ever use<br>Current	11-18 years	Y
Survey on Health and Use on Call-Up and Preparation for Defense Day (ESCAPAD)[9]	France	2017	39115	Population-based sampling.	M: 50%<br>F: 50%	Pen-and-paper	Ever use<br>Current<br>Daily	17-18 years	N, superseded by later survey
Die Drogenaffinität Jugendlicher in der Bundesrepublik Deutschland[10]	Germany	2019	2735	Random sampling	M: 52%<br>F: 48%	Telephone	Ever use<br>Current	12-17 years	Y
Rauchen bei Jugendlichen und jungen Erwachsenen in Deutschland (Alkoholsurvey)[11]	Germany	2018	2755	Weighted sampling applied to adjust for representativeness	M: 52%<br>F: 48%	Telephone	Ever use<br>Current	18-19 years	N, superseded by later survey
The decline of cigarette smoking among adolescents and young adults in Germany and the rising relevance of waterpipes, e-cigarettes and e-hookahs [12]	Germany	2016	2459	Weighting sampling applied for representativeness	M: 51%<br>F: 49%	Telephone	Current	12-17 years	N, superseded by later survey
The German Study on Tobacco Use (DEBRA)[13]	Germany	2016-2017	446	Stratified random sampling.	NR10	Face-to-face	Ever use<br>Current	14-17 years	N, superseded by later survey

The use of e-cigarettes-a population-based cross-sectional survey of 4002 individuals in 2016[14]	Germany	2016	330	Random sampling.	NR	Telephone	Ever use<br>Current	14-19 years	N, superseded by later survey
Thematic Household Survey Hong Kong – School Based Survey[15]	Hong Kong, Special Administrative Region of China	2018-2019	51700	Probability sampling.	NR	Pen-and-paper	Ever use<br>Current<br>Daily<br>Occasional	9-17 years	Y
Youth in Iceland research (Ungt 8olk) by The Icelandic Centre for Social Research and Analysis (ICSRA)[16]	Iceland	2020	10593	Population-based sampling.	NR	NR	Ever use	13-16 years	Y
Heat-not-burn tobacco, electronic cigarettes, and combustible cigarette use among Japanese adolescents: a nationwide population survey 2017 [17]	Japan	2017	64152	Cluster sampling. Weighting applied	M: 50%<br>F: 50%	Pen-and-paper	Ever use<br>Current<br>Daily	12-18 years	Y
Current tobacco and e-cigarette use among adolescents in Malaysia (TECMA)[a18]	Malaysia	2016	13162	Two stage stratified cluster sampling design.	NR	Pen-and-paper	Ever use<br>Current<br>Daily<br>Occasional<br>ENDS only<br>ENNDS only	10-19 years	N, superseded by later survey
National Health and Morbidity Survey – Adolescent Health Survey[19]	Malaysia	2017	27497	Cluster-based sampling.	M: 50%<br>F: 50%	Pen-and-paper	Current	13-17 years	Y
National Survey on Consumption of Drugs, Alcohol and Tobacco (ENCODAT)[20]	Mexico	2016	12081	Cluster-based sampling across 32 states in Mexico.	M: 51%<br>F: 49%	Face-to-face	Ever use<br>Current	12-17 years	Y
ASH Year 10 NZ[21]	NZ	2019	27354	Population-based sampling.	NR	Pen-and-paper	Ever use<br>Daily<br>Occasional	14-15 years	Y
New Zealand Health Survey (NZHS)[b22]	NZ	2018-2019	NR	Probability sampling.	NR	Mixed	Ever use<br>Current<br>Daily	15-17 years	N, superseded by later survey and no denominator
The Youth Insights Survey[a23]	NZ	2018	2689	Probability sampling.	M: 49%<br>F: 51%	Pen-and-paper	Ever use<br>Current<br>Daily	14-15 years	N, superseded by later survey
Korea Youth Risk Behavior Web-based Survey[24]	Republic of Korea	2018	60040	Cluster-based sampling.	M: 51%<br>F: 49%	Online	Current	13-18 years	Y
Scottish Schools Adolescent Lifestyle and Substance Use Survey (SALSUS)[25]	Scotland (United Kingdom)	2018	23365	Cluster based	NR	Mixed	Ever use<br>Current (Use > once per week)	13 – 15 years	Y
Encuesta sobre uso de drogas en enseñanzas secundarias en España (ESTUDES)[26]	Spain	2019	38010	Cluster-based sampling.	NR	Pen-and-paper	Ever use<br>Current<br>Ever use ENDS only and ENNDS only	14-18 years	Y
Alcohol and drug use among students (Skolelevers drogvanor) CAN school survey [27]	Sweden	2019	9876	Weighted sampling applied for representativeness	NR	Mixed	Ever use	15-19 years	Y
Action on Smoking and Health (ASH) Smokefree Great Britain Youth survey [28]	UK	2019	2523	Weighted sampling applied for representativeness	NR	Online	Ever use<br>Current<br>Occasional	11-18 years	Y

							Current ENDS only and ENNDS only		
UK Household Longitudinal Study[29]	UK	2015-2017	3291	Cluster-based sampling.	NR	Mixed	Ever use	10-15 years	N, superseded by later survey
Monitoring the Future[30]	USA	2019	13802	Multi-stage random sampling. One third of the sample received the vaping questions.	NR	Mixed	Ever use<br>Current<br>Daily ENDS only and ENNDS only	13-18 years	Y
National Youth Tobacco Survey[31]	USA	2019	18934	Probability sampling.	M: 53%<br>F: 47%	Online	Ever use ENDS only<br>Current<br>Frequent<br>Daily	11-18 years	Y
Negative health symptoms reported by youth e-cigarette users: Results from a national survey of US youth [32]	USA	2017	975	Probability-sampling.	M: 50%<br>F: 50%	Telephone	Ever use<br>Current	13-17 years	N, superseded by later survey
Population Assessment of Tobacco and Health (PATH) study[33]	USA	2016-2017	14516	Probability-based sampling.	NR	Face-to-face	Ever use<br>Current	12-17 years	N, superseded by later survey
Prevalence of Awareness and Use of JUUL E-cigarettes in a National probability Sample of adolescents in the United States [34]	USA	2018	610	Random sampling.	NR	Online	Ever use<br>Current<br>Frequent<br>Infrequent<br>Former	13-17 years	N, superseded by later survey
Truth Longitudinal Cohort[35]	USA	2019	3536	Probability-based sampling.	NR	Mixed	Ever use<br>Current	15-20 years	Y
Youth Risk Behavior Survey (YRBS)[36]	USA	2019	13205	Cluster-based sampling	M: 49%<br>F: 51%	Pen-and-paper	Ever use<br>Current<br>Daily<br>Frequent	15-18 years	Y
Childhood Exposure to Environmental Tobacco Smoke (CHETS) Wales 3 survey [37]	Wales (United Kingdom)	2019	2218	Probability sampling.	M: 51%<br>F: 49%	NR	Ever use	9-11 years	Y
Global Youth Tobacco Survey[c38]	Antigua and Barbuda, Argentina, Bolivia, Cambodia, Chile, Colombia, Cook Islands, Croatia, Cuba, Czech Republic, Dominican Republic, Ecuador, Fiji, Georgia, Ghana, Grenada, Guam, Italy, Jamaica, Kosovo, Kuwait, Lao People's Democratic Republic, Malta, Marshall Islands, Mauritius, Morocco, North Macedonia, Panama, Papua New	2016-2019	611-60914	Cluster-based sampling.	M: 38% - 50%<br>F: 50% - 62%	Pen-and-paper	Ever use<br>Current<br>Daily<br>Occasional	11-17 years	Y

	Guinea, Poland, Qatar, Romania, Saint Lucia, Serbia, Slovakia, Slovenia, Samoa, Suriname, Taiwan, Tunisia, Ukraine, Vanuatu								
Health Behaviour in School-aged Children[d39]	Austria [40], Denmark[41], Estonia[42], Greece[43], Iceland[44], Ireland [45], Kazakhstan[46b], Lithuania[47], Scotland[48], Sweden[49], Switzerland[50], Wales[51]	2018	1310-103971	Cluster based	M: 42·7%-50·1%<br>F: 49·9%-57·3%	Mixed	Ever use<br>Current<br>Daily	11-17 years	Y

[a]Not included in meta-analysis but included in narrative synthesis of type of ENDS/ENNDS
[b]Data extracted for inclusion in meta-analysis but insufficient data to pool prevalence
[c]42 GYTS all included in the pooled analysis
[d]12 HBSC surveys 8 included in the analysis
M=Male, F=Female, NR=Not reported, NZ=New Zealand, UK=United Kingdom, USA=United States of America

Table S2. Use of Electronic Non-Nicotine Delivery Systems only by frequency of reported use

Frequency of use	Subgroup	N	Prevalence (95% CI)
**Ever use**	Canada	62850	28·8 [28·5, 29·2]
	Italy	1518	18·4 [16·5, 20·4]
	Spain	38010	32·5 [32·0, 32·5]
	United States of America	13802	25·3 [24·6, 26·0]
**Current use**$	Canada	62850	11·3 [11·1, 11·6]
	United Kingdom	2523	0·9 [0·6, 1·4]
	United States of America	13802	9·6 [9·1,10·1]
**Occasional use**&	Canada	62580	9·3 [9·1, 9·6]
**Daily use**	Canada	62850	2·0 [1·9, 2·1]
	United States of America	13802	2·0 [1.8, 2·2]

$Current use included: use in last 30 days or self-reported current users

&Occasional use included: less than weekly, but more than once in the last 30 days; and less than daily, but at least once a week

Table S3. Use of Electronic Nicotine Delivery Systems only by frequency of reported use

Frequency of use	Subgroup	N	Prevalence (95% CI)
**Ever use**	Austria	10279	40.0 [39.1, 41.0]
	Canada	62850	28.1 [27.8, 28.5]
	Italy	1518	6.4 [5.3, 7.7]
	Spain	38010	5.5 [5.2, 5.7]
	United States of America	32736	33.7 [31.5, 33.1]
**Current use**[$]	Austria	10279	13.0 [12.4, 13.7]
	Canada	62850	17.7 [17.4, 18.0]
	United Kingdom	2523	3.3 [2.6, 4.0]
	United States of America	32736	19.1 [17.5,18.7]
**Occasional use**[&]	Canada	62580	10.8 [10.5, 11.0]
**Daily use**	Canada	62850	7.0 [6.8, 7.2]
	United States of America	13802	9.2 [8.7, 9.7]

[$]Current use included: use in last 30 days or self-reported current users
[&]Occasional use included: less than weekly, but more than once in the last 30 days; and less than daily, but at least once a week

Table S4. Risk of bias for eligible studies

Author/Study Name, Year	Sample frame appropriate	Participants recruited appropriately	Adequate sample size	Subjects and setting described in detail	Sufficient coverage of sample (in analysis)	Valid methods to identify condition	Condition measured in a standard, reliable way for all participants	Appropriate statistical analysis	Response rate adequate or managed appropriately if low
National Drug Strategy Household Survey, 2019	Low Risk	Low Risk	Low Risk	High Risk	Low Risk	Unclear	Unclear	Low Risk	Low Risk
ESPAD, 2019[b]	Low Risk	Low Risk	Low Risk	Low Risk	Low Risk	Unclear	Low Risk	Unclear	Low Risk
Canadian Student Tobacco, Alcohol and Drugs Survey, 2018 - 2019	Low Risk	Low Risk	Low Risk	Unclear	Low Risk	Unclear	Low Risk	Low Risk	Unclear
Health Survey for England, 2018	Low Risk	Low Risk	Low Risk	Low Risk	Low Risk	Unclear	Low Risk	Low Risk	Low Risk
Smoking, Drinking and Drug Use Amongst Young People, 2018	Low Risk	Low Risk	Low Risk	High Risk	Low Risk	Unclear	Low Risk	Low Risk	Low Risk
National School Health Promotion Survey, 2019	Low Risk	Low Risk	Low Risk	Unclear	Unclear	Unclear	Low Risk	Unclear	Unclear
Die Drogenaffinität Jugendlicher in der Bundesrepublik Deutschland, 2019	Low Risk	Low Risk	Low Risk	Low Risk	Low Risk	Unclear	Low Risk	Low Risk	Unclear
Thematic Household Survey Hong Kong, 2018-2019	Low Risk	Low Risk	Low Risk	High Risk	Unclear	Unclear	Low Risk	Unclear	High Risk
Youth in Iceland, 2019	Low Risk	Low Risk	Low Risk	High Risk	Low Risk	Unclear	Low Risk	Low Risk	Low Risk
Kuwabara, 2017 - 2018	Low Risk	Low Risk	Low Risk	Low Risk	Low Risk	Unclear	Low Risk	Low Risk	Low Risk
Tobacco and E-Cigarette Survey Among Malaysian Adolescents (TECMA), 2016[a]	Low Risk	Low Risk	Low Risk	Low Risk	Unclear	Low Risk	Low Risk	Unclear	Low Risk
National Health and Morbidity Survey, 2017	Low Risk	Low Risk	Low Risk	Low Risk	Low Risk	Low Risk	Low Risk	Low Risk	Low Risk
ENCODAT, 2017	Low Risk	Low Risk	Low Risk	Low Risk	Low Risk	Unclear	Low Risk	Low Risk	Low Risk
ASH Year 10 NZ, 2019	Low Risk	Low Risk	Low Risk	Low Risk	High Risk	Unclear	Low Risk	Low Risk	Low Risk
The Youth Insights Survey, 2018[a]	Low Risk	Low Risk	Low Risk	Low Risk	Low Risk	Unclear	Low Risk	Low Risk	Low Risk
KYRBWS, 2018	Low Risk	Low Risk	Low Risk	Low Risk	Low Risk	Unclear	Low Risk	Low Risk	Low Risk
Scottish Schools Adolescent Lifestyle and Substance Use Survey (SALSUS), 2018	Low Risk	Low Risk	Low Risk	Low Risk	Low Risk	Unclear	Low Risk	Low Risk	Low Risk
ESTUDES, 2018 - 2019	Low Risk	Low Risk	Low Risk	Low Risk	Low Risk	Unclear	Low Risk	Unclear	Low Risk
CAN school survey, 2019	Low Risk	Unclear	Low Risk	High Risk	Low Risk	Unclear	Unclear	Unclear	Unclear
Action on Smoking and Health (ASH) Smokefree	Low Risk	High Risk	Low Risk	High Risk	Low Risk	Unclear	Unclear	Low Risk	Unclear

Great Britain Youth Survey, 2019									
Monitoring the Future, 2019	Low Risk	Low Risk	Low Risk	Unclear	Low Risk	Unclear	Low Risk	Low Risk	Low Risk
US National Youth Tobacco Survey, 2019	Low Risk	Low Risk	Low Risk	Low Risk	Low Risk	Low Risk	Unclear	Low Risk	Low Risk
Truth Longitudinal Cohort (TLC), 2018	Low Risk	Unclear	Low Risk	Low Risk	Low Risk	Unclear	Unclear	Low Risk	Low Risk
Youth Risk Behavior Survey (YRBS), 2017	Low Risk	Low Risk	Low Risk	Low Risk	Low Risk	Unclear	Low Risk	Low Risk	Low Risk
Moore et al, 2020	Low Risk	Low Risk	Low Risk	Unclear	Low Risk	Unclear	Low Risk	Low Risk	Unclear
Global Youth Tobacco Survey, 2016 - 2018	Low Risk	Low Risk	Unclear	Low Risk	Low Risk	Unclear	Low Risk	Low Risk	Low Risk
HBSC Survey, 2018[b]	Low Risk	Unclear	Unclear	Low Risk	Low Risk	Unclear	Low Risk	Low Risk	Unclear

[a]Included in narrative synthesis only

[b]These two surveys combined to create the EnCLASS survey

References

1. Guerin N, White V. ASSAD 2017 Statistics & Trends: Australian Secondary Students' Use of Tobacco, Alcohol, Over-the-counter Drugs, and Illicit Substances 2020.
2. Australian Institute of Health and Welfare. National Drug Strategy Household Survey 2019. Canberra AIHW, 2020.
3. Group E. ESPAD Report 2019: Results from the European School Survey Project on Alcohol and Other Drugs. Publications Office of the European Union, Luxembourg, 2019.
4. Government of Canada. Canadian Tobacco, Alcohol and Drugs Survey (CTADS): summary of results for 2017. 2019. https://www.canada.ca/en/health-canada/services/canadian-tobacco-alcohol-drugs-survey/2017-summary.html (accessed 5th November 2020.
5. Population Health Team. NHS Digital. Health Survey for England 2018 Children's health. United Kingdom: Health and Social Care Information Centre, 2019.
6. Lifestyles Team. ND. Smoking, Drinking and Drug Use among Young People in England: 2018, 2019.
7. The Department of Health and Welfare (THL). Welfare of children and young people - School Health Survey 2019, 2019.
8. Spilka. S GE, Le Nézet. O, Ehlinger. V, Janssen. E, Brissot. A, Philippon A, Chyderiotis. S. Alcohol, tobacco and cannabis use among French school students in 2018, 2019
9. Chyderiotis S, Benmarhnia T, Beck F, Spilka S, Legleye S. Does e-cigarette experimentation increase the transition to daily smoking among young ever-smokers in France? *Drug Alcohol Depend* 2020; **208**: 107853.
10. Orth B, Merkel C. The Drug Affinity of Young People in the Federal Republic of Germany 2019. Smoking, alcohol use, and illegal drug use: current prevalence and trends. Cologne: Federal Center for Health Education, 2020.
11. Orth BM, C. . Smoking in adolescents and young adults Germany. Results of the 2018 alcohol survey and trends. BZgA research report. : Cologne: Federal Center for Health Education., 2019.
12. Orth BM, C. The decline of cigarette smoking among adolescents and young adults in Germany and the rising relevance of waterpipes, e-cigarettes and e-hookahs. *Bundesgesundheitsblatt Gesundheitsforschung Gesundheitsschutz* 2018; **61**(11): 1377-87.
13. Kotz D, Bockmann M, Kastaun S. The Use of Tobacco, E-Cigarettes, and Methods to Quit Smoking in Germany. *Dtsch Arztebl Int* 2018; **115**(14): 235-42.
14. Eichler M, , Blettner, M,. Singer, S. The Use of E-Cigarettes A Population-Based Cross-Sectional Survey of 4002 Individuals in 2016. *Dtsch Arztebl Int* 2016; **113**: 847–54.
15. Census and Statistics Department HKSAR. Thematic Household Survey - Report No. 70. Pattern of Smoking. Hong Kong, 2020.
16. Kristjansson AL, Allegrante JP, Sigfusson J, Sigfusdottir ID. Do population trends in adolescent electronic cigarette use coincide with changes in prevalence of cigarette smoking? *Prev Med Rep* 2019; **15**: 100913.
17. Kuwabara Y, Kinjo A, Fujii M, et al. Heat-not-burn tobacco, electronic cigarettes, and combustible cigarette use among Japanese adolescents: a nationwide population survey 2017. *BMC Public Health* 2020; **20**(1): 741.
18. Institute for Public Health (IPH). Tobacco & E-Cigarette Survey Among Malaysian Adolescents (TECMA). Kuala Lumpur, 2016.
19. Institute for Public Health (IPH). National Health and Morbidity Survey (NHMS) 2017: Adolescent Health Survey Malaysia, 2017.
20. Zavala-Arciniega L, Reynales-Shigematsu LM, Lozano P, Rodríguez-Andrade MÁ, Arillo-Santillán E, Thrasher JF. Patterns of awareness and use of electronic cigarettes in Mexico, a middle-income country that bans them: Results from a 2016 national survey. *Prev Med* 2018; **116**: 211-8.
21. ASH Action for Smokefree 2025 Group. ASH Year 10 Snapshot Vaping and E-cigarettes, 2019.
22. Ministry of Health. Methodology Report 2018/19: New Zealand Heath Survey: Wellington: Ministry of Health., 2019.
23. Lucas N, Gurram N, Thimasarn-Anwar T. Smoking and vaping behaviours among 14 and 15-year-olds: Results from the 2018 Youth Insights Survey.: Wellington: Health Promotion Agency/Te Hiringa Hauora Research and Evaluation Unit, 2020.
24. Kim Y, Choi S, Chun C, Park S, Khang YH, Oh K. Data Resource Profile: The Korea Youth Risk Behavior Web-based Survey (KYRBS). *Int J Epidemiol* 2016; **45**(4): 1076-e.
25. Black C, Hutcheson L. Scottish Schools Adolescent Lifestyle and Substance Use Survey (SALSUS). Technical Report (2018). Scotland: Ipsos MORI 2018.

26. Spanish Observatory on Drugs and Addictions Government Delegation for the National Plan on Drugs Secretary of State for Social Services. Survey on Drug Use in Secondary Education in Spain (ESTUDES) Encuesta sobre uso de drogas en enseñanzas secundarias en España (ESTUDES). 2019.
27. Central Association for Alcohol and Drug Information. School students' drug habits. Stockholm, 2019.
28. ASH Action on smoking and health group. Use of e-cigarettes among young people in Great Britain, 2019.
29. Green MJ, Gray L, Sweeting H, Benzeval M. Socioeconomic patterning of vaping by smoking status among UK adults and youth. *BMC Public Health* 2020; **20**(1): 183.
30. Johnston LD, Miech, R. A., O'Malley, P. M., Bachman, J. G., Schulenberg, J. E., & Patrick, M. E. Monitoring the Future national survey results on drug use 1975-2018: Overview, key findings on adolescent drug use: University of Michigan, 2019.
31. Cullen KA, Gentzke AS, Sawdey MD, et al. e-Cigarette Use Among Youth in the United States, 2019. *JAMA* 2019; **322**(21): 2095-103.
32. King JL, Reboussin BA, Merten JW, Wiseman KD, Wagoner KG, Sutfin EL. Negative health symptoms reported by youth e-cigarette users: Results from a national survey of US youth. *Addict Behav* 2020; **104**: 106315.
33. Rostron BL, Cheng YC, Gardner LD, Ambrose BK. Prevalence and Reasons for Use of Flavored Cigars and ENDS among US Youth and Adults: Estimates from Wave 4 of the PATH Study, 2016-2017. *Am J Health Behav* 2020; **44**(1): 76-81.
34. McKeganey NR, C. Prevalence of Awareness and Use of JUUL E-cigarettes in a National Probability Sample of Adolescents in the United States. *Am J Health Behav* 2019; **43**: 591-605.
35. Vallone DMC, A. F.; Briggs, J.; Xiao, H.; Schillo, B. A.; Hair, E. C. Electronic Cigarette and JUUL Use Among Adolescents and Young Adults. *JAMA Pediatr* 2020; **21**.
36. Creamer MR, Everett Jones S, Gentzke AS, Jamal A, King BA. Tobacco Product Use Among High School Students - Youth Risk Behavior Survey, United States, 2019. *MMWR Suppl* 2020; **69**(1): 56-63.
37. Moore GF, Angel L, Gray L, et al. Associations of Socioeconomic Status, Parental Smoking and Parental E-Cigarette Use with 10-11-Year-Old Children's Perceptions of Tobacco Cigarettes and E-Cigarettes: Cross Sectional Analysis of the CHETS Wales 3 Survey. *Int J Environ Res Public Health* 2020; **17**(3).
38. World Health Organization. Global youth tobacco survey (GYTS). https://www.who.int/tobacco/surveillance/gyts/en/2020).
39. Inchley J, Currie D, Cosma A, Samdal O. Health Behaviour in School-aged Children (HBSC) Study Protocol: background, methodology and mandatory items for the 2017/18 survey. St Andrews: CAHRU, 2018.
40. Felder-Puig. R TF, Ramelow. D, Maier. G,. Health and health behavior by Austrian school children. Results of the WHO-HBSC-Survey 2018. Vienna, 2019.
41. The National Institute of Public Health. The school children survey 2018. Health, well-being and health behavior among 11-, 13- and 15-year-old school students in Denmark (HBSC). Copenhagen, 2018.
42. Oja L PJ, Aasvee K, Haav A, Kasvandik L, Kukk M, Kukke K, Rahno J, Saapar M, Vorobjov S. Estonian schoolchildren's health behavior 2017/2018 academic year survey report (HBSC), 2019.
43. Kokkevi A. F, A., Kanavou, E., Stavrou, M. Smoking in adolescence. Series of topics Issues: Adolescents, Behaviors & Health (HBSC). Athens: University Mental Health Research Institute, Of Neuroscience and Medical Precision 2019.
44. Laboratory of Leisure Studies at the University of Iceland's School of Education. Health and living conditions of school students. Iceland. (HBSC), 2018.
45. Költő A GA, Molcho M, Kelly C, Walker L, Gabhainn S N. The Irish Health Behaviour in School-aged Children (HBSC) Study 2018, 2020.
46. Abdrakhmanova S, Adayeva A, Slazhneva T. Factors influencing health and well-being of children and adolescents in Kazakhstan. Key findings from the Health Behavior in School-Aged Children (HBSC) Study in the Republic of Kazakhstan, 2017., 2019.
47. Šmigelskas K LJ, Slapšinskaitė A, Vaičiūnas T, Bulotaitė J, Žemaitaitytė M, Šalčiūnaitė A, Zaborskis A. Lithuanian students' lifestyle and health: 2018 situation and trends HBSC study. Kaunas, 2019.
48. Inchley J, Mokogwu D, Mabelis J, Currie D. Health Behaviour in School-aged Children (HBSC) 2018 Survey in Scotland: National Report: University of Glasgow, 2020.
49. The Swedish Public Health Agency. School children's health habits in Sweden 2017/18 (HBSC). Stockholm, 2019.
50. Delgrande JM, Schneider E, Eichenberger Y, Kretschmann A, Schmidhauser V, Masseroni S. Eating habits, activity physical, weight status and body image in students aged 11 to 15 Switzerland - Results of the "Health Behavior in School-aged Children" survey (HBSC) 2018 and evolution over time 2020.
51. Hewitt G. AR, Moore G., Melendez-Torres G.J., Murphy S. . Student Health and Wellbeing In Wales: Report of the 2017/18 Health Behaviour in School-aged Children Survey and School Health (HBSC). Cardiff, UK: Cardiff University, 2019.
